# Anti-Proliferative Effect of an Aqueous Extract of *Prunella vulgaris* in Vascular Smooth Muscle Cells

**DOI:** 10.1155/2013/936463

**Published:** 2013-09-12

**Authors:** Sun Mi Hwang, Yun Jung Lee, Yong Pyo Lee, Jung Joo Yoon, So Min Lee, Jeong Dan Cha, Kyung Min Choi, Dae Gill Kang, Ho Sub Lee

**Affiliations:** ^1^College of Oriental Medicine and Professional Graduate School of Oriental Medicine, Wonkwang University, Shinyong-dong, Iksan, Jeonbuk 570-749, Republic of Korea; ^2^Center for Bioanalysis, Division of Metrology for Quality of Life, Korea Research Institute of Standards and Science, Doryong-dong, Yuseong-gu, Daejeon 305-340, Republic of Korea; ^3^Hanbang Body-Fluid Research Center, Wonkwang University, Shinyong-dong, Iksan, Jeonbuk 570-749, Republic of Korea; ^4^Department of Research Development, Institute of Jinan Red Ginseng, Jinan, Jeonbuk 567-801, Republic of Korea

## Abstract

The abnormal proliferation of vascular smooth muscle cells (VSMCs) in arterial walls is an important pathogenic factor of vascular disorders such as diabetic atherosclerosis. We have reported the anti-inflammatory effect of an aqueous extract from *Prunella vulgaris* (APV) in vascular endothelial cell. In the present study, APV exhibited inhibitory effects on high glucose-stimulated VSMC proliferation, migration, and invasion activities, inducing G_1_ cell cycle arrest with downregulation of cyclins and CDKs and upregulation of the CKIs, p21^waf1/cip1^ and p27^kip1^. Furthermore, APV dose dependently suppressed the high glucose-induced matrix metalloproteinase activity. High glucose-induced phosphorylation of ERK, p38 MAPK, was decreased by the pretreatment of APV. NF-**κ**B activation by high glucose was attenuated by APV, as an antioxidant. APV attenuated the high glucose-induced decrease of nuclear factor E2-related factor-2 (Nrf2) translocation and heme oxygenase-1 (HO-1) expression. Intracellular cGMP level was also increased by APV treatment. These results demonstrate that APV may inhibit VSMC proliferation via downregulating ROS/NF-**κ**B /ERK/p38 MAPK pathways. In addition, APV has a beneficial effect by the interaction of Nrf2-mediated NO/cGMP with HO-1, suggesting that *Prunella vulgaris* may be useful in preventing diabetic atherosclerosis.

## 1. Introduction

Atherosclerosis is one of the most important pathogenic mechanisms in vascular dysfunction. Chronic hyperglycemia-induced atherosclerosis involves a complex series of events, including abnormal vascular smooth muscle cells (VSMCs) proliferation and migration, which contribute importantly to the formation of organized atherosclerotic plaque [1, 2]. VSMCs are the main cellular component of the arterial wall and fundamental for the maintenance of normal vascular function and structure. Within the arterial media, VSMCs are normally arrested at G_0_/G_1_ phase of the cell cycle and are thus quiescent [3]. After vessel injury by inflammation and oxidative stress, VSMCs migrate into the intima, where they reenter the cell cycle [4]. Transition through G_1_ phase and entry into S phase require activation of cyclin-dependent kinases (CDKs) such as CDK2 and CDK4 through the formation of cyclin/CDK complexes, a process in which cyclin D1 and cyclin E play major roles. The kinase activities of the cyclin/CDK complexes are negatively regulated by CDK inhibitors (CKIs) such as p21^waf1/cip1^ and p27^kip1^ [5, 6]. High glucose activates the expression of several genes involved in mitogen-activated protein kinase- (MAPK-) dependent mitogenic response, contributing to VSMC proliferation and migration, as a result, to the development of atherosclerosis [7]. VSMC migration, which depends on an alteration of the proteolytic balance within the arterial wall toward matrix breakdown, is partly mediated by matrix metalloproteinase (MMP) [8–10]. The promoter region of MMP-2 contains various cis-acting elements, including potential binding sites for the transcription factors: nuclear factor-kappa B (NF-*κ*B), activator protein-1 (AP-1), and stimulatory protein-1 (SP-1). Various growth factors, cytokines, and hormones stimulate the expression of MMP-2 via activation of the NF-*κ*B pathway [11, 12]. 

Heme oxygenase- (HO-) 1 is a stress-inducible protein well known for playing a role of important components in intracellular antioxidant, anti-inflammatory, and antiapoptotic effects [13, 14]. HO-1, which is highly expressed in vascular tissues, protects against vasculopathy and confers a cytoprotective function in the circulation. HO-1 deficiency results in cytokines production-mediated endothelial damage [15]. Therefore, HO-1 may play an important role in sustaining the health of the vascular system. Nuclear factor erythroid 2-related factor 2 (Nrf2) is a redox-sensitive transcription factor that normally resides in the cytoplasm bound to Kelch-like ECH-associated protein- (Keap-) 1 [16]. Upon activation by oxidative stress, it binds to the antioxidant response element (ARE) and activates transcription of ARE-regulated genes. ARE-regulated genes may contribute to the maintenance of redox homeostasis by serving as endogenous antioxidant systems through the action of proteins such as heme oxygenase-1 (HO-1), ferritin, glutathione peroxidase (GPx), and NAD(P)H: quinone oxidoreductase [17].

*Prunella vulgaris *var. lilacina (herbal name: *Prunellae Spica*) is a perennial herb that is widely distributed around Far East Asia countries throughout Korea, China, and Japan. *Prunella vulgaris* has been used as a traditional medicine to reduce sore throat, alleviate fever, and accelerate wound healing. In addition to dried flower, fruit-spike of *Prunella vulgaris* has been used in oriental medicine to treat hypertension and tuberculosis [18, 19]. A variety of components (campherol, rutin, triterpenoids, and phenolic acids; rosmarinic acid, caffeic acid, and tannins) have been identified [20]. We previously reported the anti-inflammatory and antidiabetic effects of *Prunella vulgaris* [19, 21]. Now, we investigated the anti-proliferative effect of an aqueous extract from *Prunella vulgaris* (APV) on high glucose stimulating human aortic smooth muscle cells (HASMCs). 

## 2. Methods and Materials

### 2.1. Preparation of an Aqueous Extract of *Prunella vulgaris*

The *Prunella vulgaris *var. lilacina (Herba) was purchased from Herbal Medicine Cooperative Association in Jeollabuk Province, Republic of Korea, in January 2010. A voucher specimen (no. HBN161) has been deposited in the Herbarium of the Professional Graduate School of Oriental Medicine, Wonkang University (Korea). The dried *Prunella vulgaris* var. lilacina (100 g) was soaked for 2 hr in water (1 L) and then boiled in distilled water at 100°C for 2 hr. The yield of aqueous extract of *Prunella vulgaris* (APV) was approximately 16.27% of the plant powder. The extract was subsequently concentrated using rotary evaporator and then used in the present study.

### 2.2. Cell Culture

HASMCs were obtained from Cascade Biologics (Van Allen Way, Carlsbad, CA), as cryopreserved primary cultures, and grew in culture flask in vascular smooth muscle cell growth medium; M231 was supplemented with 5% smooth muscle growth supplement (SMGS) and 0.2% gentamicin/amphotericin solution according to Cascade Biologics' recommended protocol. Cells of passages 5 and 6 were grown in monolayers at 37°C, in a humidified atmosphere of 5% CO_2_ and 95% air, and used for experiments at >80% confluence.

### 2.3. Determination of Cell Proliferation

[^3^H]-thymidine incorporation was performed to determine effect of APV on high glucose-induced cell proliferation in HASMCs [22]. Briefly, HASMCs on the 24-well culture plates were pretreated with or without APV for 1 h, which was followed by treatment with high glucose at 37°C for 24 h. After incubation, 1 *μ*Ci of [^3^H]-thymidine (PerkinElmer, Boston, MA) was added for 24 h. The medium was removed, and the cells were washed with ice-cold PBS. Next, plates were incubated with 10% TCA at room temperature for 5 min and then solubilized at room temperature for 30 min in 0.3 N NaOH, 1% SDS. [^3^H]-thymidine activity was measured in liquid scintillation counter (LS 6500 Multipurpose Scintillation Counter, Beckman, Fullerton, CA).

### 2.4. Cell Migration Assay

The cell migration assay was evaluated by wound healing assay [23]. Briefly, HASMCs on the 12-well culture plates were incubated approximately 80% confluent and then made a scratch with yellow tip. Next, HASMCs were pretreated with APV for 1 h, which was followed by treatment with high glucose at 37°C for 24 h. After incubation, the microscopic photographs of migrated cells were measured by a fluorescence microscopy (Leica Microsystems Wetzlar GmbH, Wetzlar, Germany).

### 2.5. Cell Invasion Assay

The cell invasion assay [24] was cultured on matrigel-coated filter inserts that fit into 24-well matrigel invasion chamber obtained from BD (Two Oak Park, Bedford, MA). Briefly, HASMCs on the 24-well invasion chambers were incubated with APV for 1 h, which was followed by treatment with high glucose at 37°C for 24 h. After incubation, the cells on the upper side of the filter were removed using cotton swabs. The migrated cells were fixed by methyl alcohol for 5 min and then stained with hematoxylin. The microscopic photographs of invaded cells were measured by a fluorescence microscopy.

### 2.6. Western Blot Analysis

Cell homogenates (40 *μ*g of protein) were separated on 10% SDS-polyacrylamide gel electrophoresis and transferred to nitrocellulose paper. Blots were then washed with H_2_O, blocked with 5% skimmed milk powder in Tris-Buffered Saline Tween-20 (TBST) (10 mM Tris-HCl, pH 7.6, 150 mM NaCl, 0.05% Tween-20) for 1 h, and incubated with the appropriate primary antibody at dilutions recommended by the supplier. Then the membrane was washed, and primary antibodies were detected with goat anti-rabbit-IgG or rabbit anti-mouse-IgG conjugated to horseradish peroxidase, and the bands were visualized with enhanced chemiluminescence (Amersham Bioscience, Buckinghamshire, UK). Protein expression levels were determined by analyzing the signals captured on the nitrocellulose membranes using the ChemiDoc image analyzer (Bio-Rad Laboratories, Hercules, CA).

### 2.7. Gelatin Zymography

HASMCs were pretreated with APV for 1 h and stimulated with high glucose for 24 h. The supernatant, conditioned medium was collected for zymography [25]. SDS-PAGE for measurement of MMP-2 activity was added with 0.1% gelatin in the 10% separated gel. The gel was washed with renaturation buffer (2.5% Triton X-100 in DW) at room temperature for 1 h and then incubated with development buffer (Invitrogen Corporation, Carlsbad, CA) at 37°C for overnight. Next, the gel was stained with 0.2% Coomassie brilliant blue R stain reagent at room temperature for 1 h and then washed with destain buffer for look to visualize the clear area using a ChemiDoc image analyzer.

### 2.8. Transient Transfection and Luciferase Assay

The smooth muscle cells were grown to 60–80% confluence, and the cells were transiently cotransfected with the plasmids using Lipofectamine LTX (Invitrogen, Carlsbad, CA) according to the manufacturer's protocol [26]. Briefly, the transfection mixture containing 0.5 *μ*g of either the pGL3-4*κ*B-Luc or pGL4.MMP-9-Luc2 and 0.1 *μ*g of pCMV-*β*-gal was mixed with the Lipofectamine LTX reagent and added to the cells. After 24 h, the cells were treated with APV for 30 min and stimulated with high glucose for 24 h and then lysed. The luciferase and *β*-galactosidase activities were determined as described elsewhere using Luciferase assay kit (Promega, Madison, WI). The luciferase activity was normalized with respect to the *β*-galactosidase activity and expressed as a percentage of the activity of the high glucose group.

### 2.9. Radioimmunoassay (RIA) for Determination of cGMP Production

The cGMP production was evaluated by RIA [27]. Briefly, HASMCs on the 6 cm culture dish were incubated approximately 80% confluent and then pretreated with APV for 1 h, which was followed by treatment with high glucose at 37°C for 24 h. After incubation, the cGMP production was measured by a *γ*-counter (1480 Automatic Gamma Counter, PerkinElmer, Turku, Finland).

### 2.10. Intracellular Reactive Oxygen Species (ROS) Assay

The fluorescent probe, 5-(and-6)-chloromethyl-2′,7′-dichlorodihydro-fluorescein diacetate, acetyl ester (CM-H_2_DCFDA), was used to determine the intracellular generation of ROS by stimulation of high glucose. Briefly, the confluent HASMCs in the 24-well culture plates were pretreated with or without APV for 1 h. After removing the APV from the wells, the cells were incubated with 20 *μ*M CM-H_2_DCFDA for 1 h. The cells were stimulated with high glucose, and the fluorescence intensity was measured at an excitation and emission wavelength of 485 nm and 530 nm, respectively, using a flow cytometry on FACScalibur (BD, San Diego, CA).

### 2.11. Statistical Analysis

All the experiments were repeated at least three times. The results were expressed as a mean ± S.E., and the data were analyzed using one-way ANOVA followed by Dunnett's test or Student's *t*-test using Sigma Plot (Sigma plot for Windows, version 10.0, USA) to determine any significant differences. *P* < 0.05 was considered as statistically significant.

## 3. Results

### 3.1. Effect of APV on High Glucose-Induced HASMCs Proliferation

**Figure 1:**
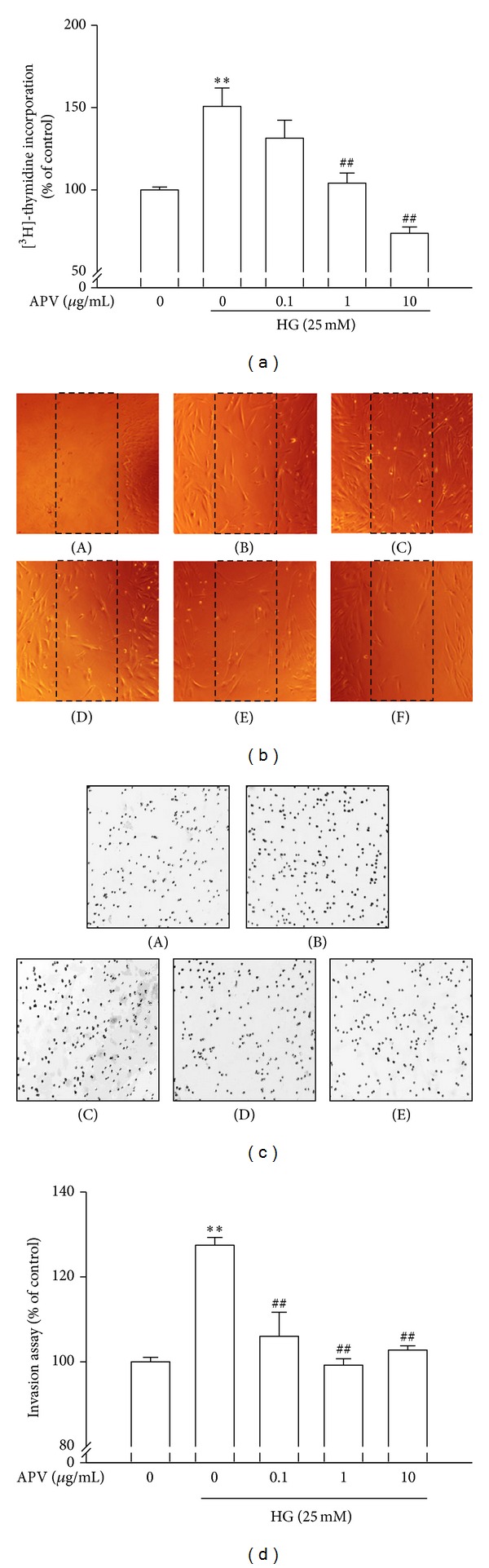
Effect of APV on high glucose-induced HASMCs proliferation, migration, and invasion. (a) Confluent HASMCs were incubated with or without APV and high glucose for 24 h and then treated with [^3^H]-thymidine for 24 h. (b) HASMCs were incubated approximately 80% confluent and then made a scratch with yellow tip. HASMCs were pretreated with APV for 1 h, which was followed by treatment with high glucose at 37°C for 24 h. ((A): wound, (B): control, (C): HG (25 mM), (D): HG + APV 0.1 *μ*g/mL, (E): HG + APV 1 *μ*g/mL, (F): HG + APV 10 *μ*g/mL). (c) HASMCs on the 24-well invasion chambers were incubated with APV for 1 h, which was followed by treatment with high glucose at 37°C for 24 h. ((A): control, (B): HG (25 mM), (C): HG + APV 0.1 *μ*g/mL, (D): HG + APV 1 *μ*g/mL, (E): HG + APV 10 *μ*g/mL). Values were expressed as mean ± S.E. of five independent experiments. (d) Each electrophoretogram is representative of the results from three individual experiments. **P < 0.01 versus control; ^##^ *P* < 0.01 versus high glucose alone.

**Figure 2:**
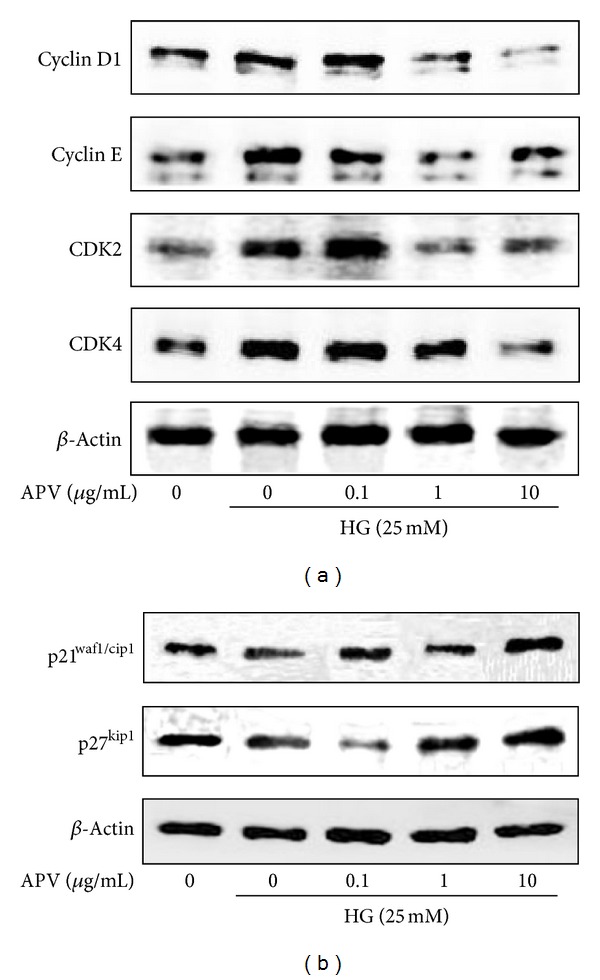
Effects of APV on high glucose-induced cell cycle regulator (a) cyclins, CDKs, and (b) CKIs proteins. The total cellular protein (40 *μ*g) extracts were prepared and separated on 10% SDS-PAGE and blotted with the antibodies specific for cyclin D1, cyclin E, CDK2, CDK4, p21^waf1/cip1^, p27^kip1^, and *β*-actin. Each electrophoretogram is representative of the results from three individual experiments.

In the [^3^H]-thymidine incorporation assay (Figure 1(a)), stimulation with high glucose (25 mM) increased DNA synthesis. Pretreatment with APV significantly decreased high glucose-induced increase of DNA synthesis in a dose-dependent manner (*P* < 0.01). To explore the anti-proliferative effect of APV, protein levels of the cyclins and CDKs were examined by Western blot analysis. As a result, pretreatment with APV decreased high glucose-induced protein expressions of cyclin D1 and cyclin E as well as CDK2 and CDK4 (Figure 2(a)). In contrast, pretreatment with APV increased high glucose-induced protein expressions of CKIs, p21^waf1/cip1^ and p27^kip1^ (Figure 2(b)). In this study, APV (1–10 *μ*g/mL) did not alter any cytotoxicity (data not shown). 

### 3.2. Effect of APV on High Glucose-Induced HASMCs Migration and Invasion

As shown in Figure 1(b), the HASMCs that migrated to the empty space were also visualized under a microscope. The HASMCs migration was increased by treatment with high glucose compared with control. However, pretreatment of APV inhibited cell migration. In addition, effect of APV in measurement of high glucose-induced HASMCs invasion was detected using a BD BioCoat Matrigel Invasion Chamber. As shown in Figures 1(c) and 1(d), the cells which invaded the lower chamber were also visualized under a fluorescence microscope. The cell invasion was increased by treatment with high glucose compared with control. However, pretreatment of APV significantly inhibited HASMCs invasion (*P* < 0.01).

### 3.3. Effect of APV on High Glucose-Induced MMP-9 Activity

**Figure 3:**
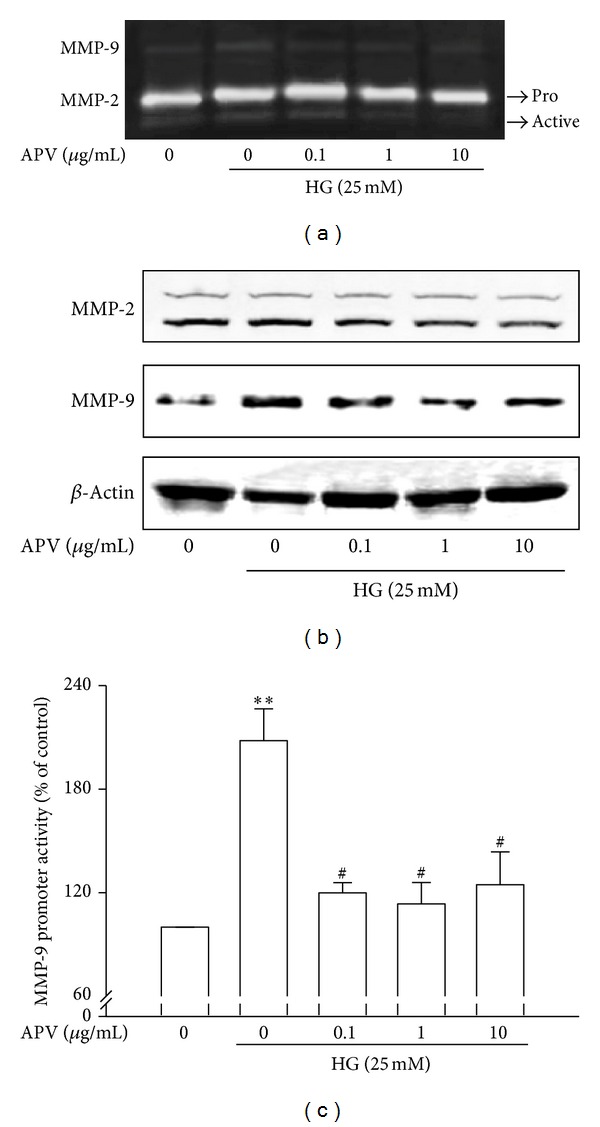
Effects of APV on high glucose-induced MMP-2 or -9 activation and expression. (a) Conditioned supernatant was prepared, and the levels of MMP-2/-9 were measured by gelatin zymography, as described in Section 2. Electrophoretogram is representative of the results from three individual experiments. (b) The total cellular protein (40 *μ*g) extracts were prepared and separated on 10% SDS-PAGE and blotted with the antibodies specific for MMP-2, MMP-9, and *β*-actin. Each electrophoretogram is representative of the results from three individual experiments. (c) The HASMCs were transiently transfected with pGL4.MMP-9-Luc2 and pCMV-*β*-gal. This was followed by harvesting, and their luciferase activities were determined. Values were expressed as mean ± S.E. of three individual experiments. **P < 0.01 versus control; ^#^ *P* < 0.05 versus high glucose alone.

The results of gelatin zymography revealed that high glucose increased MMP-9 activity as zymogen secretion without change of MMP-2 (Figure 3(a)). To determine whether APV inhibits high glucose-induced increase of MMP-9 expression, Western blot analysis was performed (Figure 3(b)). Pretreatment with APV decreased MMP-9 activation and expression induced by high glucose. Thus, transient transfections were performed using the MMP-9-dependent luciferase reporter plasmid in order to further examine the effect of APV on the MMP-9 transcription activity. As shown in Figure 3(c), high glucose increased MMP-9 transcription activity, and the concentration over 0.1 *μ*g/mL of APV significantly inhibited high glucose-induced MMP-9 transcriptional activity (*P* < 0.05). However, activation and expression of MMP-2 have no significant difference by APV treatment.

### 3.4. Effect of APV on High Glucose-Induced *NF-KappaB* Activation

**Figure 4:**
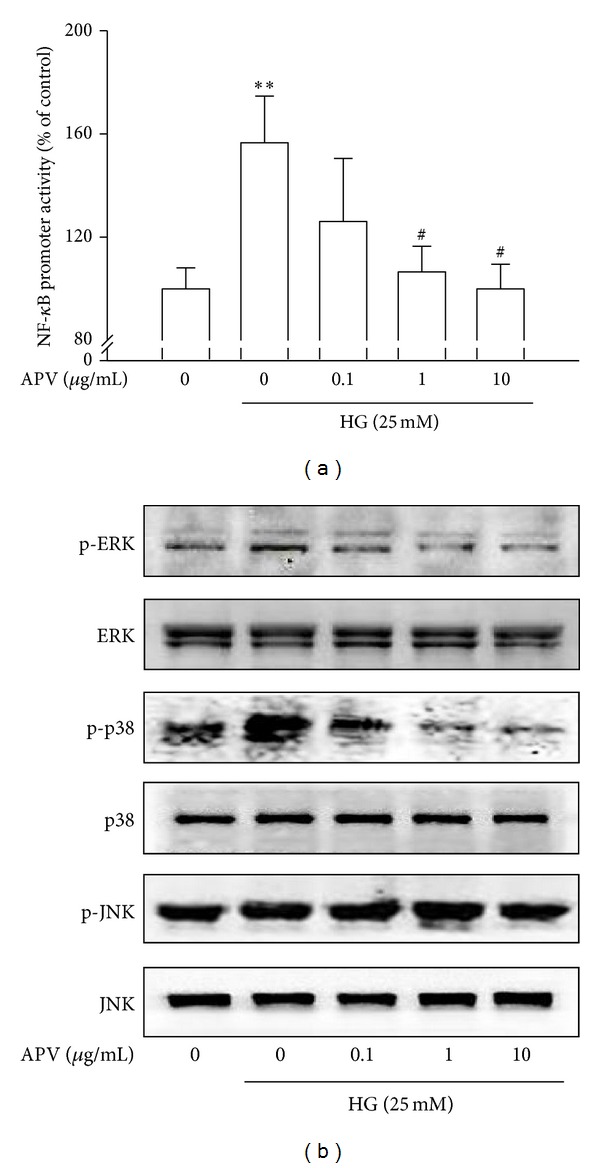
(a) Effect of APV on high glucose-induced *NF-kappaB* activation. The HASMCs were transiently transfected with pGL3-4*κ*B-Luc and pCMV-*β*-gal. This was followed by harvesting, and their luciferase activities were determined. Values were expressed as mean ± S.E. of three individual experiments. **P < 0.01 versus control; ^#^ *P* < 0.05 versus high glucose alone. (b) Effect of APV on high glucose-induced MAPKs activation. The total cellular protein (40 *μ*g) extracts were prepared and separated on 10% SDS-PAGE and blotted with the antibodies specific for p-ERK, p-p38, and p-JNK. Each electrophoretogram is representative of the results from three individual experiments.

*NF-kappaB* promoter activity measured whether APV could suppress *NF-kappaB* promoter in HASMCs. Thus, transient transfections were performed using the *NF-kappaB*-dependent luciferase reporter plasmid in order to further examine the effects of APV on the *NF-kappaB* transcription activity. As shown in Figure 4(a), high glucose increased *NF-kappaB* transcription activity, and the concentration over 1 *μ*g/mL of APV significantly inhibited high glucose-induced *NF-kappaB* transcriptional activity.

### 3.5. Effect of APV on High Glucose-Induced p-ERK and p38 MAPKs Activation

To confirm the involvement with MAPKs family in regulation of HASMCs proliferation by APV, it was performed by Western blotting. As a result, high glucose induced phosphorylations of p44/42 ERK and p38 MAPKs in HASMCs. However, APV decreased the high glucose-induced phosphorylations of ERK and p38 MAPKs. On the other hand, JNK has no significant difference in its phosphorylation (Figure 4(c)).

### 3.6. Effect of APV on Nrf2-Mediated HO-1 and cGMP Signaling Pathways

**Figure 5:**
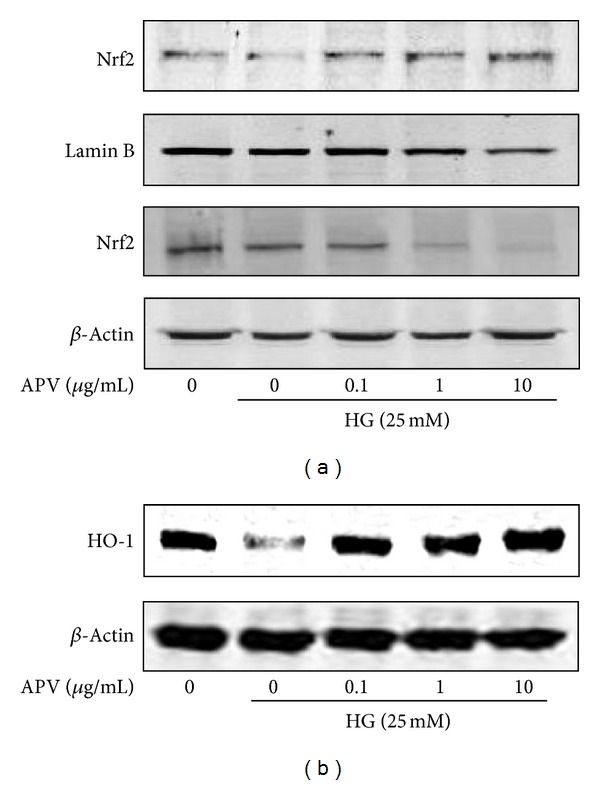
(a) Effect of APV on high glucose-induced expression of Nrf2 and translocation into nucleus in HASMCs. Cytoplasm and nuclei fractions were extracted, and protein levels were determined by Western blot analysis. Values were expressed as mean ± S.E. of three individual experiments. (b) Effect of APV on high glucose-induced expression of HO-1. The total cellular protein (40 *μ*g) extracts were prepared and separated on 10% SDS-PAGE and blotted with the antibody specific for HO-1 and *β*-actin. Each electrophoretogram is representative of the results from three individual experiments.

**Figure 6:**
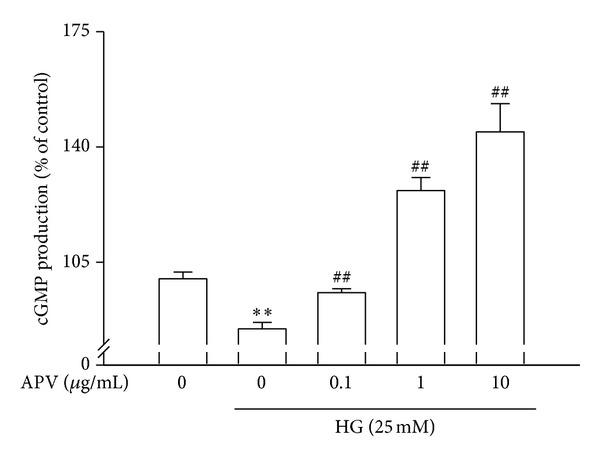
Effect of APV on high glucose-induced suppression of cGMP production in HASMCs. Values were expressed as mean ± S.E. of three individual experiments. **P < 0.01 versus control; ^##^ *P* < 0.01 versus high glucose alone.

To determine the effect of APV on the HO-1 formation, nuclear translocation of nuclear factor E2-related factor 2 (Nrf2) in HASMCs was examined.  Figure 5(a) showed that high glucose significantly decreased the translocation of Nrf2 in nuclei, whereas pretreatment with APV increased Nrf2 translocation in nuclei (Figure 5(a)). Likewise, high glucose decreased expression of heme oxygenase-1 (HO-1), but it was increased by treatment of APV (Figure 5(b)). In the experiment to determine whether APV inhibits high glucose-induced decrease of cGMP, various APV concentrations ranging from 0.1 to 10 *μ*g/mL were added to HASMCs. As shown in Figure 6, pretreatment with APV increased high glucose-induced suppression of cGMP in a dose-dependent manner (*P* < 0.01).

### 3.7. Effect of APV on High Glucose-Induced ROS Formation

**Figure 7:**
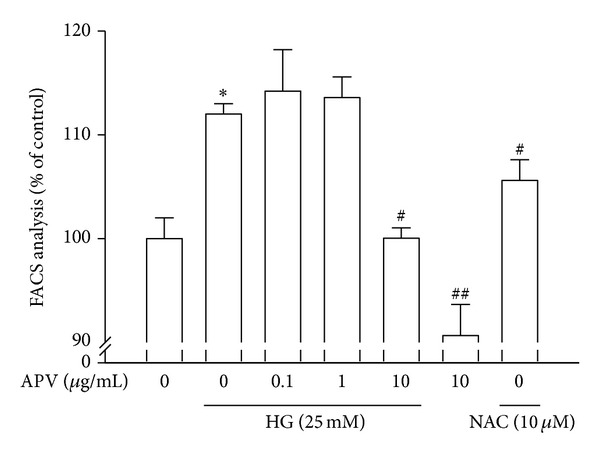
Effect of APV on high glucose-induced ROS formation. The fluorescence intensity of cells was measured using a flow cytometry on FACScalibur. Values were expressed as mean ± S.E. of five independent experiments. *P < 0.05 versus control; ^#^ *P* < 0.05, ^##^ *P* < 0.01 versus high glucose alone.

ROS has been implicated as a common second messenger in various pathways leading to NF-*κ*B activation [21]. Thus, the level of intracellular ROS production was assessed by monitoring the fluorescence in order to determine whether APV can reduce the level of high glucose-induced oxidative stress in HASMCs. APV was compared with NAC (10 *μ*M) as a positive control. As a result, DCF fluorescence level showed a marked increase after treatment with high glucose. However, pretreatment with APV and NAC significantly inhibited high glucose-induced DCF-sensitive cellular ROS levels (Figure 7).

## 4. Discussion

In arterial media, VSMCs are normally quiescent and remain in the G_0_/G_1_ phase of cell cycle. VSMC proliferation and migration play a key role in the atherosclerosis which are induced by high blood plasma concentration [1]. Thus, the aim of the present study was to determine whether APV exerts an antiatherogenic property throughout the inhibition of HASMCs proliferation. We previously reported an anti-inflammatory effect of APV on high glucose-stimulated vascular endothelial cells, suggesting a possibility of anti-proliferative effect [19, 21]. Consistent with the previous report, APV inhibited high glucose-induced HASMC proliferation in a dose-dependent manner. 

The action of MMPs has recently emerged as an important component of the natural history of atherosclerosis and of the vascular response to injury [28, 29]. Especially, gelatinase, MMP-2, and MMP-9 play pivotal roles in HASMC proliferation, and the inhibitory effect on their expression is important in the search for therapeutic natural herbs [30]. Considerable interest in this study was the marked decrease in the secretions of MMP-9 activity from high glucose-induced HASMCs in response to APV. APV decreased not MMP-2 but MMP-9 in measurement of zymography or immunoblot analysis. This result suggests that an anti-proliferative effect of APV is specific to the inhibition of MMP-9 in HASMC. Some in vitro research reported that inhibition of both cell proliferation and MMP-9 activity has appeared by various oriental herbs, which is used for the treatment of atherosclerosis [19, 31]. MMP-9 gene expression can be activated via a number of signaling pathways including those involving MAPKs family such as ERK1/2, p38 MAPK, JNK, and PI3K/Akt, which are the upstream modulators of AP-1 or NF-*κ*B [32]. Our results showed that high concentrations of glucose stimulated phosphorylation of p38 MAPKs and ERK, whereas p38 MAPKs and ERK were attenuated by a pretreatment with APV. In contrast to these two kinases, JNK is not activated in the present condition, and it was also reported in the diabetic rats or mesangial cells upon exposure to high glucose [33, 34]. However, other studies suggested the involvement of MAPKs family such as ERK, p-38, and JNK in VSMC proliferation [6, 35]. We suspect that this discrepancy is dependent on the various conditions including origin, time, and stimuli. Thus, a further study is needed to clarify a possible role of MAPKs family in response to high glucose. These observations led us to suggest the participation of p38 MAPKs and ERK in HASMC proliferation. Thus, we demonstrate that APV could ameliorate HASMC proliferation via inhibiting the activities of MMP-9, p-p38, and p-ERK.

It has been well established that high glucose-induced oxidative stress may be a key factor in the development of diabetic vascular complications [36]. Recent studies have demonstrated that the increased level of ROS is one of the most important contributors to diabetic vascular complications, which is produced as a result of longstanding hyperglycemic stress [37, 38]. Therefore, blocking the generation of excess ROS will significantly improve vascular injury in diabetics. In the present study, APV attenuated high glucose-induced ROS production as well as the activations of ERK and p38 MAPK and NF-*κ*B signaling pathways, implying that APV-mediated inhibitory effects on high glucose-stimulated HASMCs proliferation are due to blocking ROS-dependent ERK and p38 MAPK signaling cascades. These findings suggest that APV may have beneficial effect in protecting against vascular complications in diabetics.

HO, a cytoprotective enzyme, is the rate-limiting enzyme in the degradation of heme. Among the three HO isoforms reported, HO-1 is highly inducible by hemin and by a vast array of nonheme substances such as endotoxin and hydrogen peroxide, suggesting that HO-1 may play a significant role in protection of tissues from oxidative injuries [39]. It is confirmed that APV increased Nrf2 activation and HO-1 expression against high glucose condition in vascular endothelial cells [21]. The Nrf2/antioxidant response element (ARE) pathway plays an important role in regulating cellular antioxidants, including HO-1, which is a cytoprotective enzyme [40]. Thus, our findings suggest that Nrf2-mediated HO-1 upregulation was mediated by APV in adaptive survival response to high glucose-induced oxidative stress. 

Since reduced NO-cGMP signaling contributes to vascular inflammation, firstly, endothelial-derived NO production has attracted increasing attention in this study [41]. It was reported that oriental herb-mediated NO signaling is involved in endothelial protection against the HG-induced vascular inflammation [42]. Despite high glucose stimulation, APV induced intracellular cGMP production, truly having the powerful vascular relaxation effect in VSMCs. However, it could not be concluded that APV-induced cGMP production is dependent on or independent of endothelium, because there is no direct evidence that APV induced cGMP production without NO donor in the present study. Thus, a further study is required to clarify the mechanisms of oriental herb-activated endogenous defense system against vascular inflammation. 

Nitrosative stress caused by reactive nitrogen species such as NO and peroxynitrite overproduced during inflammation leads to cell death and has been implicated in the pathogenesis of many human ailments. However, relatively mild nitrosative stress may improve cellular defense capacities, rendering cells tolerant or adaptive to ongoing and subsequent cytotoxic challenges [43]. On the other hand, the fact that activated Akt-Nrf2 signaling plays a critical role in the regulation of the vasoprotective effect of APV is supported by our results. In addition, it is clear that HO-1 has been shown to be an important biological target of NO [44]. Thus, we suggest that APV-induced HO-1 activation in smooth muscle and bioactive NO in endothelium contribute to induce defense against HG-induced vascular inflammation. 

In conclusion, APV showed potent antidiabetic atherosclerosis effects on high glucose-induced vascular smooth muscle cell proliferation, possibly through the inhibition of ROS/NF-*κ*B and activation of Nrf2/HO-1 pathways. Our data provide a possible molecular mechanism mediating the inhibitive effect of APV on HASMCs proliferation. In the light of results, *Prunella vulgaris* could be an effective therapeutic candidate against diabetes-associated cardiovascular diseases.
